# Supplementary material for: Coherent dynamics of strongly interacting electronic spin defects in hexagonal boron nitride
Source: Nat Commun. 2023 Jun 6;14:3299. doi: 10.1038/s41467-023-39115-y (PMC10244381; doi:10.1038/s41467-023-39115-y)
Supplement: Supplementary file 1 — Supplementary Information [file 41467_2023_39115_MOESM1_ESM.pdf]

Supplementary Information: Coherent Dynamics of Strongly Interacting Electronic Spin Defects in Hexagonal Boron Nitride

Ruotian Gong,[1] Guanghui He,[1] Xingyu Gao,[2] Peng Ju,[2] Zhongyuan Liu,[1] Bingtian Ye,[3,4]
Erik A. Henriksen,[1,5] Tongcang Li,[2,6] Chong Zu[1,5,†]
[1]Department of Physics, Washington University, St. Louis, MO 63130, USA
[2]Department of Physics and Astronomy, Purdue University, West Lafayette, Indiana 47907, USA
[3]Department of Physics, Harvard University, Cambridge, MA 02138, USA
[4]Department of Physics, University of California, Berkeley, CA 94720, USA
[5]Institute of Materials Science and Engineering, Washington University, St. Louis, MO 63130, USA
[6]Elmore Family School of Electrical and Computer Engineering, Purdue University, West Lafayette, IN 47907, USA
[†]To whom correspondence should be addressed; E-mail: zu@wustl.edu

(Dated: May 12, 2023)

Supplementary Note 1. EXPERIMENTAL SETUP

We characterize the coherent dynamics of $\mathrm{V_B^-}$ ensemble using a home-built confocal laser microscope. A 532 nm laser (Millennia eV High Power CW DPSS Laser) is used for both $\mathrm{V_B^-}$ spin initialization and detection. The laser is shuttered by an acousto-optic modulator (AOM, G&H AOMO 3110-120) in a double-pass configuration to achieve $> 10^5 : 1$ on/off ratio. An objective lens (Mitutoyo Plan Apo 100x 378-806-3) focuses the laser beam to a diffraction-limited spot with diameter ~ 0.6 μm and collects the $\mathrm{V_B^-}$ fluorescence. The fluorescence is then separated from the laser beam by a dichroic mirror, and filtered through a long-pass filter before being detected by a single photon counting module (Excelitas SPCM-AQRH-63-FC). The signal is processed by a data acquisition device (National Instruments USB-6343). The objective lens is mounted on a piezo objective scanner (Physik Instrumente PD72Z1x PIFOC), which controls the position of the objective and scans the laser beam vertically. The lateral scanning is performed by an X-Y galvanometer (Thorlabs GVS212).

To isolate an effective two-level system $|m_s = 0, -1\rangle$, we position a permanent magnet directly on top of the sample to create an external magnetic field B $\sim$ 250 G along the c-axis of the hBN lattice. Under this magnetic field, the $|m_s = \pm 1\rangle$ sublevels of the $\mathrm{V_B^-}$ are separated due to the Zeeman effect, and exhibits a splitting $2\gamma_e B$, where $\gamma_e = 2.8$ MHz/G is the gyromagnetic ratio of the $\mathrm{V_B^-}$ electronic spin. A resonant microwave drive with frequency 2.76 GHz is applied to address the transition between $|m_s = 0\rangle \Longleftrightarrow |m_s = -1\rangle$ sublevels.

The microwave driving field is generated by mixing the output from a microwave source (Stanford Research SG384) and an arbitrary wave generator (AWG, Chase Scientific Wavepond DAx22000). Specifically, a high-frequency signal at 2.635 GHz from the microwave source is combined with a 0.125 GHz signal from the AWG using a built-in in-phase/quadrature (IQ) modulator, so that the sum frequency at 2.76 GHz is resonant with the $|m_s = 0\rangle \Longleftrightarrow |m_s = -1\rangle$ transition. By modulating the amplitude, duration, and phase of the AWG output, we can control the strength, rotation angle, and axis of the microwave pulses. The microwave signal is amplified by a microwave amplifier (Mini-Circuits ZHL-15W-422-S+) and delivered to the hBN sample through a coplanar waveguide. The microwave is shuttered by a switch (Minicircuits ZASWA-2-50DRA+) to prevent any leakage. All equipments are gated through a programmable multi-channel pulse generator (SpinCore PulseBlasterESR-PRO 500) with 2 ns temporal resolution.

We remark that in order to efficiently drive the $\mathrm{V_B^-}$ spin, the strength of the microwave pulse is set to $\Omega_\mathrm{p} = 83$ MHz in our experiment, corresponding to a $\frac{\pi}{2}$- and π-pulse length as short as 3 ns and 6 ns respectively. The AWG we use has a sampling rate 2 GHz (0.5 ns temporal resolution), sufficiently fast to generate high-fidelity pulses to control the spin state of $\mathrm{V_B^-}$ ensemble.

Supplementary Note 2. DIPOLAR HAMILTONIAN UNDER THE ROTATING-WAVE APPROXIMATION

Supplementary Note 2.1. Hamiltonian Derivation

In this section, we derive the dipolar interacting Hamiltonian of the V_B^- ensemble described by Eq. (1) from the main text. In the laboratory frame, the spin dipole-dipole interaction between two V_B^- defects can be written as:

$$\mathcal{H}_{\text{dip}} = -\frac{J_0}{r^3}[3(\hat{\mathcal{S}}_1 \cdot \hat{n})(\hat{\mathcal{S}}_2 \cdot \hat{n}) - \hat{\mathcal{S}}_1 \cdot \hat{\mathcal{S}}_2], \tag{1}$$

where $J_0 = 52$ MHz·nm^3, r and $\hat{n}$ denote the distance and direction unit vector between two V_B^- centers, and $\hat{\mathcal{S}}_1$ and $\hat{\mathcal{S}}_2$ are the V_B^- spin-1 operators. Our experiments only focus on an effective two-level system $\{|m_s = 0\rangle, |m_s = -1\rangle\}$, so the spin operators in the restricted Hilbert space are:

$$\mathcal{S}^z = \begin{bmatrix} 0 & 0 \\ 0 & -1 \end{bmatrix}, \ \mathcal{S}^x = \frac{1}{\sqrt{2}}\begin{bmatrix} 0 & 1 \\ 1 & 0 \end{bmatrix}, \ \mathcal{S}^y = \frac{1}{\sqrt{2}}\begin{bmatrix} 0 & -i \\ i & 0 \end{bmatrix}. \tag{2}$$

Also, we can define the spin raising and lowering operators:

$$\mathcal{S}^+ = \begin{bmatrix} 0 & 1 \\ 0 & 0 \end{bmatrix} = \frac{\mathcal{S}^x + i\mathcal{S}^y}{\sqrt{2}}, \ \mathcal{S}^- = \begin{bmatrix} 0 & 0 \\ 1 & 0 \end{bmatrix} = \frac{\mathcal{S}^x - i\mathcal{S}^y}{\sqrt{2}}, \tag{3}$$

and rewrite spin operators in terms of the raising and lowering operators:

$$\mathcal{S}^x = \frac{\mathcal{S}^+ + \mathcal{S}^-}{\sqrt{2}}, \ \mathcal{S}^y = \frac{\mathcal{S}^+ - \mathcal{S}^-}{i\sqrt{2}}. \tag{4}$$

Then we can expend the dipolar interaction in Supplementary Eq. (1) as:

$$\begin{aligned} \mathcal{H}_{\text{dip}} = -\frac{J_0}{r^3} \times \Bigg\{ 3 \left[\mathcal{S}_1^z n_z + \frac{(\mathcal{S}_1^+ + \mathcal{S}_1^-)n_x}{\sqrt{2}} + \frac{(\mathcal{S}_1^+ - \mathcal{S}_1^-)n_y}{i\sqrt{2}} \right] \left[\mathcal{S}_2^z n_z + \frac{(\mathcal{S}_2^+ + \mathcal{S}_2^-)n_x}{\sqrt{2}} + \frac{(\mathcal{S}_2^+ - \mathcal{S}_2^-)n_y}{i\sqrt{2}} \right] \\ - \left[\mathcal{S}_1^z \mathcal{S}_2^z + \frac{(\mathcal{S}_1^+ + \mathcal{S}_1^-)}{\sqrt{2}} \frac{(\mathcal{S}_2^+ + \mathcal{S}_2^-)}{\sqrt{2}} + \frac{(\mathcal{S}_1^+ - \mathcal{S}_1^-)}{i\sqrt{2}} \frac{(\mathcal{S}_1^+ - \mathcal{S}_1^-)}{i\sqrt{2}} \right] \Bigg\}. \end{aligned} \tag{5}$$

For each V_B^- center, there is a splitting $\Delta = 2.76$ GHz between the two levels $|m_s = 0\rangle$ and $|m_s = -1\rangle$ along the z direction (under a external magnetic field ~ 250 G). Therefore, the evolution driven by $\Delta\mathcal{S}^z$ is worth to be noted. Consider a quantum state $|\phi\rangle$ in the rotating frame $|\varphi\rangle = e^{-i\Delta\mathcal{S}^z t}|\phi\rangle$. If we apply Schrödinger equation:

$$\begin{aligned} i\partial_t|\varphi\rangle &= (\Delta\mathcal{S}^z + \mathcal{H}_{\text{dip}})|\varphi\rangle \\ i\partial_t(e^{-i\Delta\mathcal{S}^z t}|\phi\rangle) &= (\Delta\mathcal{S}^z + \mathcal{H}_{\text{dip}})(e^{-i\Delta\mathcal{S}^z t}|\phi\rangle) \\ \Delta\mathcal{S}^z e^{-i\Delta\mathcal{S}^z t}|\phi\rangle + e^{-i\Delta\mathcal{S}^z t} i\partial_t|\phi\rangle &= \Delta\mathcal{S}^z e^{-i\Delta\mathcal{S}^z t}|\phi\rangle + \mathcal{H}_{\text{dip}} e^{-i\Delta\mathcal{S}^z t}|\phi\rangle \\ i\partial_t|\phi\rangle &= e^{i\Delta\mathcal{S}^z t}\mathcal{H}_{\text{dip}} e^{-i\Delta\mathcal{S}^z t}|\phi\rangle. \end{aligned} \tag{6}$$

Then we can define dipolar interaction Hamiltonian in the rotating frame:

$$\tilde{\mathcal{H}}_{\text{dip}} = e^{i\Delta\mathcal{S}^z t} \cdot \mathcal{H}_{dip} \cdot e^{-i\Delta\mathcal{S}^z t}, \tag{7}$$

and the spin operators in the rotating frame:

$$\begin{aligned} \tilde{\mathcal{S}}^z &= e^{i\Delta\mathcal{S}^z t} \cdot \mathcal{S}^z \cdot e^{-i\Delta\mathcal{S}^z t} = \mathcal{S}^z \\ \tilde{\mathcal{S}}^+ &= e^{i\Delta\mathcal{S}^z t} \cdot \mathcal{S}^+ \cdot e^{-i\Delta\mathcal{S}^z t} = \mathcal{S}^+ \cdot e^{+i\Delta t} \\ \tilde{\mathcal{S}}^- &= e^{i\Delta\mathcal{S}^z t} \cdot \mathcal{S}^- \cdot e^{-i\Delta\mathcal{S}^z t} = \mathcal{S}^- \cdot e^{-i\Delta t}. \end{aligned} \tag{8}$$

In the rotating frame, rewrite the dipolar interaction Hamiltonian (Supplementary Eq. (5)):

$$\tilde{\mathcal{H}}_{\rm dip} = -\frac{J_0}{r^3}\times\left\{3\left[\tilde{\mathcal{S}}_1^z n_z + \frac{(\tilde{\mathcal{S}}_1^+ + \tilde{\mathcal{S}}_1^-)n_x}{\sqrt{2}} + \frac{(\tilde{\mathcal{S}}_1^+ - \tilde{\mathcal{S}}_1^-)n_y}{i\sqrt{2}}\right]\left[\tilde{\mathcal{S}}_2^z n_z + \frac{(\tilde{\mathcal{S}}_2^+ + \tilde{\mathcal{S}}_2^-)n_x}{\sqrt{2}} + \frac{(\tilde{\mathcal{S}}_2^+ - \tilde{\mathcal{S}}_2^-)n_y}{i\sqrt{2}}\right] - \left[\tilde{\mathcal{S}}_1^z\tilde{\mathcal{S}}_2^z + \frac{(\tilde{\mathcal{S}}_1^+ + \tilde{\mathcal{S}}_1^-)}{\sqrt{2}}\frac{(\tilde{\mathcal{S}}_2^+ + \tilde{\mathcal{S}}_2^-)}{\sqrt{2}} + \frac{(\tilde{\mathcal{S}}_1^+ - \tilde{\mathcal{S}}_1^-)}{i\sqrt{2}}\frac{(\tilde{\mathcal{S}}_2^+ - \tilde{\mathcal{S}}_2^-)}{i\sqrt{2}}\right]\right\}, \tag{9}$$

which can be simplified to

$$\begin{aligned}\tilde{\mathcal{H}}_{dip} = -\frac{J_0}{r^3}\times\{ &(3n_z^2-1)\mathcal{S}_1^z\mathcal{S}_2^z + (\mathcal{S}_1^+\mathcal{S}_2^- + \mathcal{S}_1^-\mathcal{S}_2^+)\left[\frac{3}{2}(n_x^2+n_y^2)-1\right] \\ &+ \frac{3}{2}\mathcal{S}_1^+\mathcal{S}_2^+e^{+2i\Delta t}(n_x^2-n_y^2-2in_xn_y) + \frac{3}{2}\mathcal{S}_1^-\mathcal{S}_2^-e^{-2i\Delta t}(n_x^2-n_y^2+2in_xn_y) \\ &+ 3\mathcal{S}_1^z n_z\left[\frac{n_x}{\sqrt{2}}(\mathcal{S}_2^+e^{+i\Delta t}+\mathcal{S}_2^-e^{-i\Delta t}) + \frac{n_y}{i\sqrt{2}}(\mathcal{S}_2^+e^{+i\Delta t}-\mathcal{S}_2^-e^{-i\Delta t})\right] \\ &+ 3\mathcal{S}_2^z n_z\left[\frac{n_x}{\sqrt{2}}(\mathcal{S}_1^+e^{+i\Delta t}+\mathcal{S}_1^-e^{-i\Delta t}) + \frac{n_y}{i\sqrt{2}}(\mathcal{S}_1^+e^{+i\Delta t}-\mathcal{S}_1^-e^{-i\Delta t})\right]\}.\end{aligned} \tag{10}$$

Since we are interested in spin-spin interaction dynamics with energy scale $J_0/r^3 \approx 1.8$ MHz that is much smaller than the splitting $\Delta \approx 2.76$ GHz, we are able to drop the last six time-dependent terms and only keep the energy-conserving terms under the rotating-wave approximation. Additionally, considering $n_x^2+n_y^2+n_z^2=1$, we get

$$\begin{aligned}\tilde{\mathcal{H}}_{\rm dip} &= -\frac{J_0}{r^3}\times(3n_z^2-1)[\mathcal{S}_1^z\mathcal{S}_2^z - \frac{1}{2}\mathcal{S}_1^+\mathcal{S}_2^- - \frac{1}{2}\mathcal{S}_1^-\mathcal{S}_2^+] \\ &= -\frac{J_0}{r^3}\times\frac{(3n_z^2-1)}{2}[2\mathcal{S}_1^z\mathcal{S}_2^z - \mathcal{S}_1^x\mathcal{S}_2^x - \mathcal{S}_1^y\mathcal{S}_2^y].\end{aligned} \tag{11}$$

We can rewrite the interacting Hamiltonian using normal spin-$\frac{1}{2}$ operators

$$S^z = \frac{1}{2}\begin{bmatrix}1 & 0\\ 0 & -1\end{bmatrix},\; S^x = \frac{1}{2}\begin{bmatrix}0 & 1\\ 1 & 0\end{bmatrix},\; S^y = \frac{1}{2}\begin{bmatrix}0 & -i\\ i & 0\end{bmatrix}. \tag{12}$$

Specifically, we convert the effective two-level spin-1 operators to spin-$\frac{1}{2}$ operators, $\mathcal{S}^x = \sqrt{2}S^x$, $\mathcal{S}^y = \sqrt{2}S^y$, $\mathcal{S}^z = S^z + 1/2$, and plug them into Supplementary Eq. (11),

$$\mathcal{H}_{\rm dip} = -\frac{J_0\mathcal{A}}{r^3}(S_1^zS_2^z - S_1^xS_2^x - S_1^yS_2^y), \tag{13}$$

where $\mathcal{A} = 3n_z^2 - 1$ is the angular dependent factor.

To derive the dipolar Hamiltonian of the entire V_B^- spin ensemble, we simply sum up the interactions between every pair of V_B^- spins:

$$\mathcal{H}_{\rm dip} = -\sum_{i<j}\frac{J_0\mathcal{A}_{i,j}}{r_{i,j}^3}(S_i^zS_j^z - S_i^xS_j^x - S_i^yS_j^y), \tag{14}$$

where $\mathcal{A}_{i,j}$ and $r_{i,j}$ represent the angular dependence of the long-range dipolar interaction and the distance between the ith and j^{th} V_B^- centers.

Supplementary Note 2.2. T_1 Independence from Dipolar Interaction

We can also re-write the interaction Hamiltonian Supplementary Eq. (14) using raising and lowering operators, S_i^+ and S_i^-,

$$\mathcal{H}_{\rm dip} = \sum_{i<j} -\frac{J_0\mathcal{A}_{i,j}}{r_{i,j}^3}(S_i^zS_j^z - \frac{1}{2}[S_i^+S_j^- + S_i^-S_j^+]). \tag{15}$$

From this form, we can see that dipolar interaction can lead to spin flip-flop between two nearby V_B^- ($|m_s = 0\rangle \otimes |m_s = -1\rangle \iff |m_s = -1\rangle \otimes |m_s = 0\rangle$). However, when measuring ensemble T_1, we characterize dynamics of total spin polarization across the entire V_B^- ensemble, $\Sigma_i \langle S_i^z \rangle$, which remains unchanged under dipolar flip-flop. Therefore, T_1 is not expected to have a dependence on V_B^- concentration ρ. Our experimental observation of T_1 decreasing with increasing ion dosages may be attributed to the presence of lattice damage during the implantation process or local charge state hopping [1].

Supplementary Note 3. SWEEPING PULSE INTERVAL VERSUS SWEEPING PULSE NUMBER IN T_2 MEASUREMENT

To measure the coherent timescales, T_2^{XY8} and T_2^{D}, we choose to fix the time interval between pulses to be $\tau_0 = 4$ ns, much smaller than the correlation time of the noise environment, and increase the number of pulses for each subsequent data point. Importantly, the purpose of this method is two-fold: (1) By fixing the time interval between pulses, the center frequency of the noise filter function of the applied sequence is fixed during the measurement. Consequently, this allows us to sweep the measuring pulse sequence length while avoiding hitting the unwanted resonances due to the hyperfine coupling between V_B^- and the nearby nuclear spin bath; (2) Given the short-lived coherence of V_B^- , it is also crucial to obtain enough data points at the early timescale to capture the decoherence decay profiles. By fixing τ_0 to a small value, we can collect more points at the beginning to better characterize the coherent timescales.

For comparison with the fixed pulse interval method in the main text, we have also performed measurements of XY-8 on sample S_3 by fixing the pulse number at $N_0 = 8$ and $N_0 = 16$ while increasing the pulse intervals (see Supplementary Fig. 1). We observe that, for both cases, the XY-8 coherent timescale is shorter than the XY-8 measurement with a fixed pulse interval. This is not surprising as we expect the XY-8 sweep τ timescale to approach the XY-8 sweep N timescale at a large enough pulse number N_0. However, at $N_0 = 16$, the first data point of the decay profile (corresponding to $\tau = 2$ ns) is already at 128 ns due to the finite duration of the pulses, which is on the same order of the decay timescale one extract from the fitting. Therefore, we choose to fix the pulse interval time and increase the number of pulses for measuring the coherent dynamics of V_B^- throughout this work.

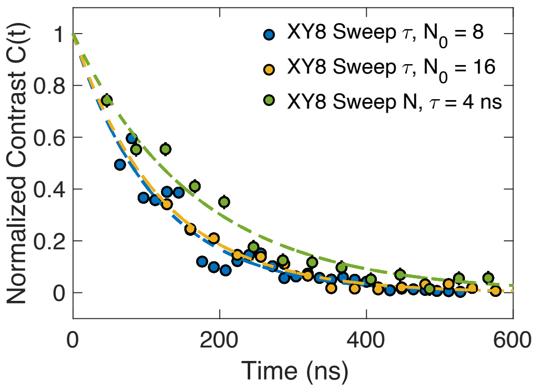

Supplementary Fig. 1. T_2^{XY8} **Comparison** XY-8 coherent timescales measured on sample S3 with the highest ion implantation dosage using three different methods. The first two are to fix the XY-8 pulse number at $N_0 = 8$ and $N_0 = 16$ while increasing the pulse intervals. The third method is to fix the time interval between pulses to be $\tau_0 = 4$ ns while increasing the number of pulses, and this is the measurement technique we choose to use. Here dashed lines are data fitting with single exponential decays. Error bars represent 1 s.d. accounting statistical uncertainties.

Sample	S1	S2	S3
$\rho_{\mathrm{V_B^-}}$ Extracted from Experiment (ppm)	123^{+8}_{-8}	149^{+25}_{-21}	236^{+35}_{-31}
Counts N ($\times 10^6$ photons/s)	0.64 ± 0.03	0.84 ± 0.03	1.44 ± 0.04
Contrast C (%)	4.74 ± 0.27	3.89 ± 0.19	4.00 ± 0.17
Adjusted Counts N_A (arb. units)	1.00 ± 0.06	1.07 ± 0.06	1.90 ± 0.11

Supplementary Table 1. Summary of the estimated $\mathrm{V_B^-}$ concentration from coherent measurement, fluorescence counts, and contrasts for the three hBN samples

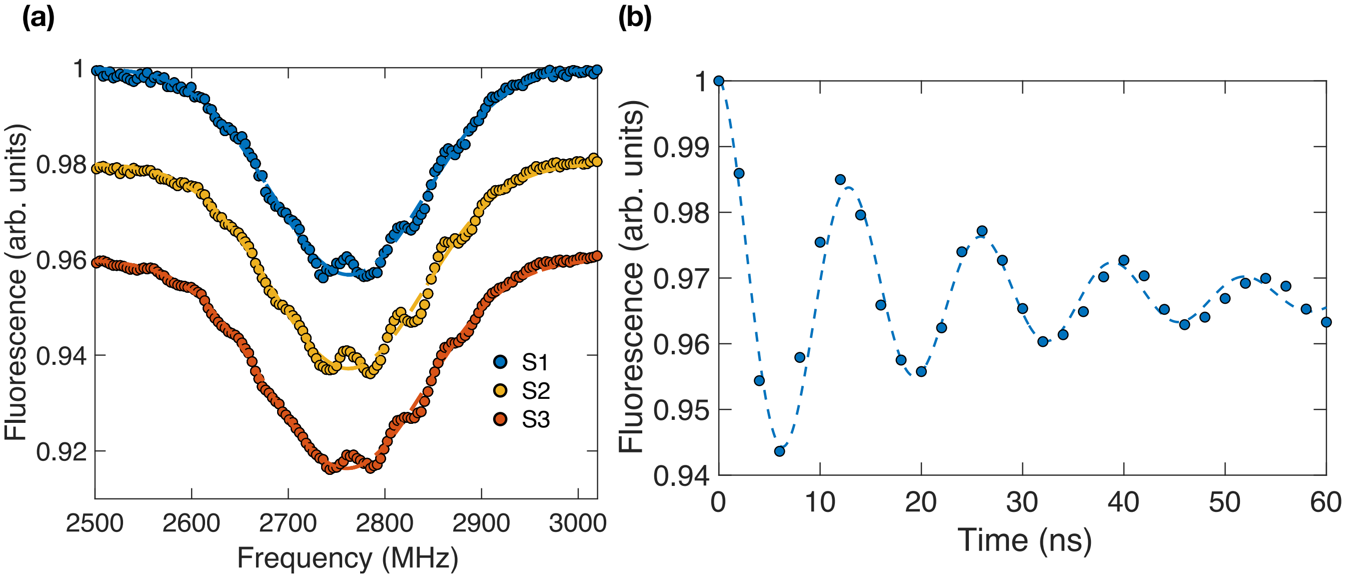

Supplementary Fig. 2. **ESR Spectrum and Rabi Oscillation** (a) Measured ESR spectra of three samples under an external magnetic field ~ 250 G. Dashed lines are the data fittings to a single Gaussian distribution. Note that fluorescences are shifted for comparison. (b) Rabi oscillation of sample S3. The dashed line is the data fitting to a shifted cosine function with a stretched exponential decay envelope.

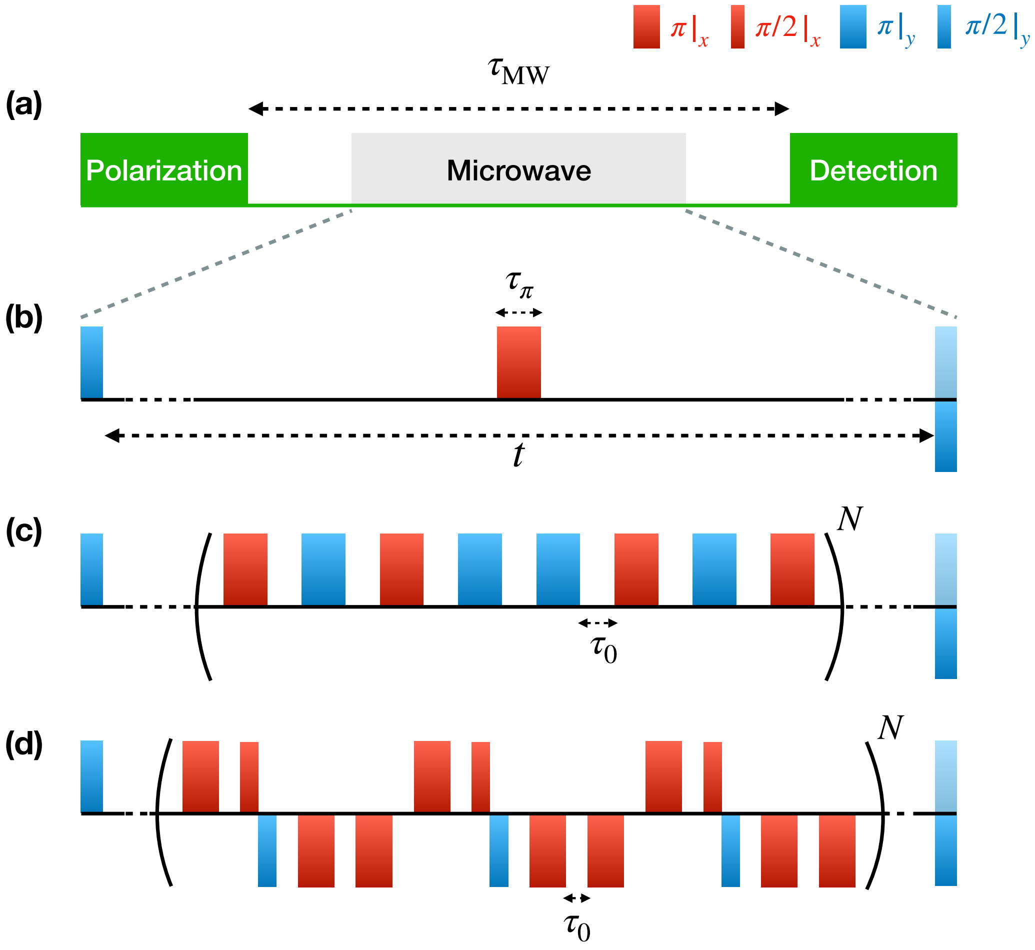

Supplementary Fig. 3. **Dynamical Decoupling Sequences** a) Sequence schematic for laser and microwave. The duration between polarization and detection, τ_{MW}, is fixed around 3500 ns to account for the effect of T_1 relaxation on the T_2 measurement. b) Pulse sequence for spin echo. The rotations along the positive $\mathbf{x}$ and $\mathbf{y}$ axes are plotted above the line, while the rotations along the negative axes are plotted below the line. π-pulse duration, τ_π, is fixed at 6 ns with adjusted microwave power referencing to the Rabi oscillation recorded Fig. 2. Here we sweep the evolution time t to measure the coherent timescale. The final $\frac{\pi}{2}$ pulse along the $\mp\mathbf{y}$ axis is applied for differential measurement. c) Pulse sequence for XY-8. The sequence repeats itself every 8 pulses, and we take a measurement every 4 pulses to increase the number of data points. The interval between every adjacent pulse, τ_0 is fixed at 4 ns. We extract the timescale by increasing the number of sequences, N. d) Pulse sequence for DROID. Here we adopt a truncated version of the original DROID sequence to increase the total number of data points [2]. τ_0 is also fixed at 4 ns, but note that there is no interval between the two adjacent $\frac{\pi}{2}$ pulses. N is swept to measure the DROID coherent timescales.

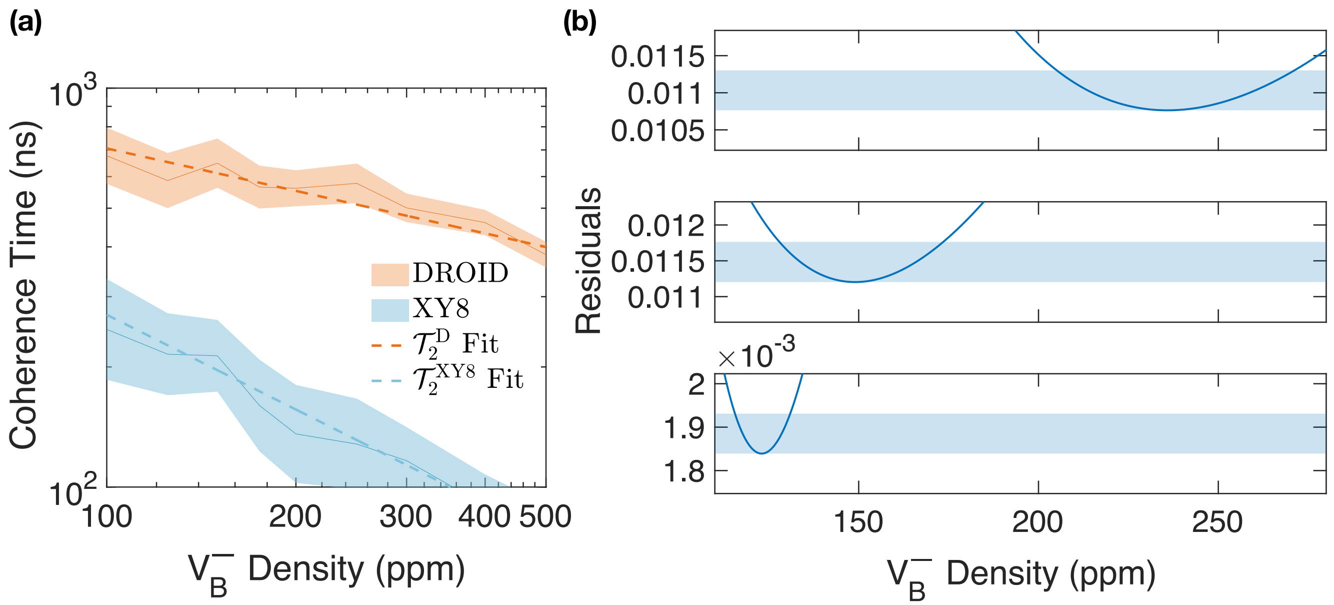

Supplementary Fig. 4. **Residuals** (a) Numerically simulated coherent timescales, $\mathcal{T}_2$, for DROID and XY-8 pulse sequences. The solid lines show the timescales extracted from simulations with error bars plotted as semi-transparent color areas. Here we fit $\mathcal{T} \propto \rho^{-\alpha}$. (b) The sum of squared relative residuals of XY-8 and DROID between the experimental values and the fitted $\mathcal{T}$ (Eqn. 5) plotted against V_B^- densities used in simulations. From bottom to top are residuals for sample S_1, S_2, and S_3 respectively, and the blue shaded regions are the 5% error ranges from the minimum residuals.

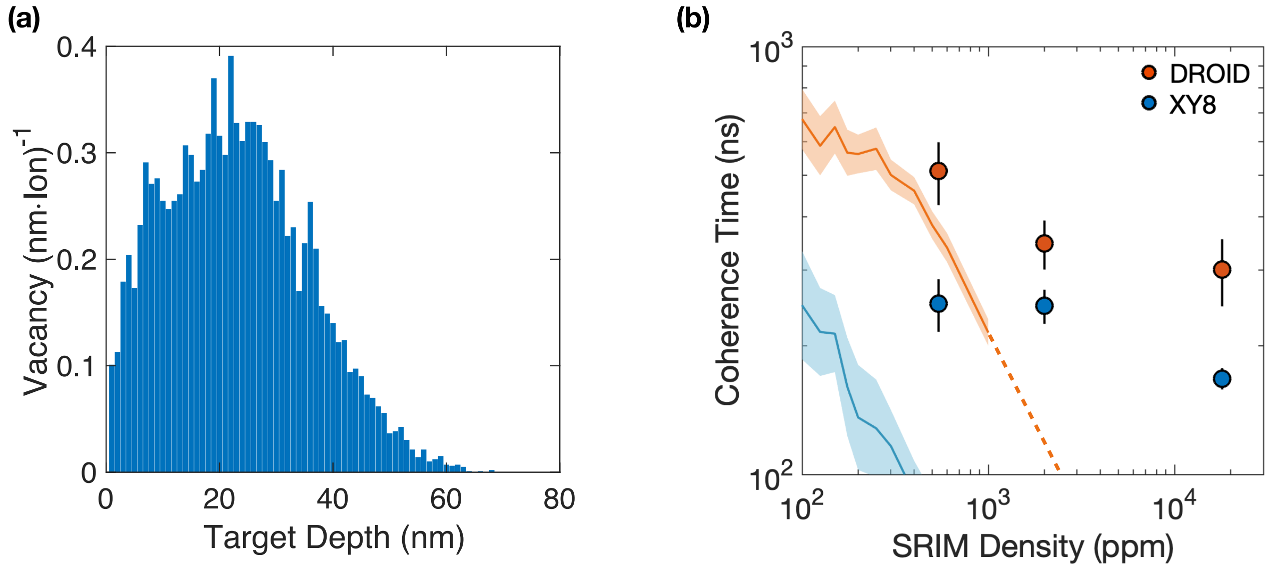

Supplementary Fig. 5. **SRIM Simulation Result** (a) SRIM simulation result for irradiating Helium ion (3 keV) on hBN layer with thickness 100 nm. The simulated boron vacancies per ion is plotted against the depth from the hBN sample surface. (b) Comparison between the experimentally measured and numerically simulated coherent timescales, T_2, for DROID and XY-8 pulse sequences. The solid lines show the timescales extracted from simulations with error bars plotted as semi-transparent color areas. Densities for experimental points are from SRIM simulation instead of estimated from coherent dynamics as in the main text. The error bars in coherence time account for 1 s.d. of fitting error, and error bars of V_B^- densities represent the range of densities whose residuals lie within 5%.

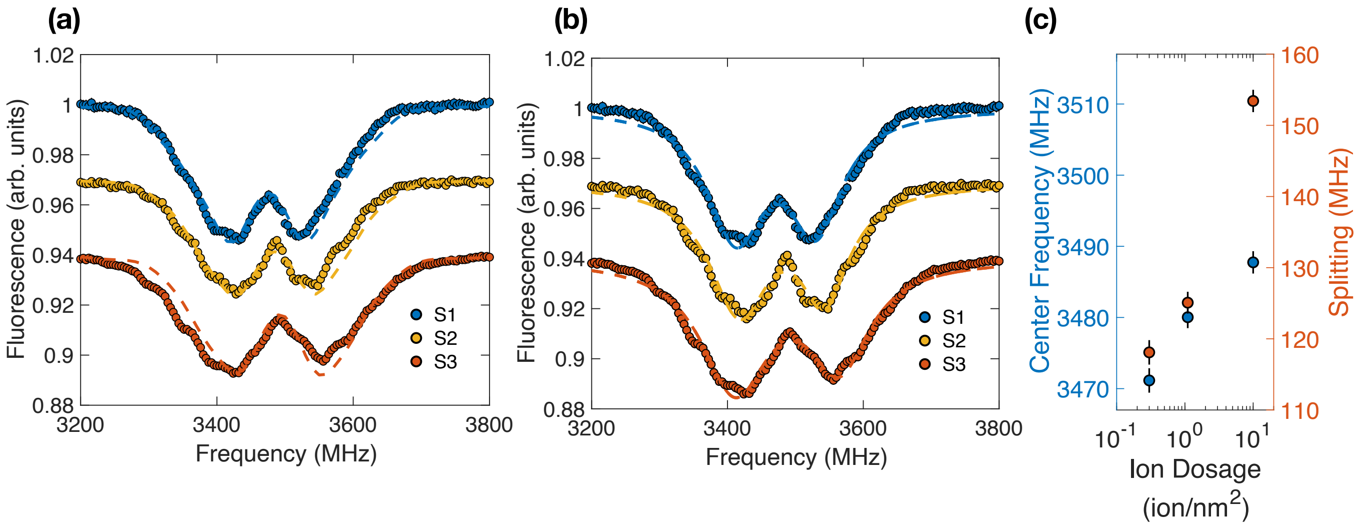

Supplementary Fig. 6. **ESR without external magnetic field** (a) Measured ESR spectra of sample S1, S2 and S3 at zero magnetic field. Dashed lines are the simulation results from our microscopic charged model using $d_\perp = 40$ Hz/(V $\cdot$ cm^{-1}) and extracted V_B^- densities $\rho_{V_B^-} = \{123^{+8}_{-8},\ 149^{+25}_{-21},\ 236^{+35}_{-31}\ \}$ ppm. (b) Dashed lines are data fittings to the sum of two Lorentzian distributions. Fluorescences are shifted vertically for comparison. (c) The ESR Splittings and Center Frequencies of three hBN samples extracted from fitting. Aside from increasing splittings, we notice a small shift of the ESR center frequencies with increasing implantation dosages, which may originate from the implantation-induced strain effect. Error bars represent 1 s.d. in fitting error.

[1] J. Choi, S. Choi, G. Kucsko, P. C. Maurer, B. J. Shields, H. Sumiya, S. Onoda, J. Isoya, E. Demler, F. Jelezko, *et al.*, Physical review letters **118**, 093601 (2017).
[2] J. Choi, H. Zhou, H. S. Knowles, R. Landig, S. Choi, and M. D. Lukin, Physical Review X **10**, 031002 (2020).
